# Bioinformatics Data Mining Repurposes the JAK2 (Janus Kinase 2) Inhibitor Fedratinib for Treating Pancreatic Ductal Adenocarcinoma by Reversing the *KRAS* (Kirsten Rat Sarcoma 2 Viral Oncogene Homolog)-Driven Gene Signature

**DOI:** 10.3390/jpm10030130

**Published:** 2020-09-16

**Authors:** Li-Wei Liu, Yao-Yu Hsieh, Pei-Ming Yang

**Affiliations:** 1 School of Nutrition and Health Sciences, Taipei Medical University, Taipei 11031, Taiwan; leo200931@gmail.com; 2 Division of Hematology and Oncology, Taipei Medical University Shuang Ho Hospital, New Taipei City 23561, Taiwan; alecto39@gmail.com; 3 Division of Hematology and Oncology, Department of Internal Medicine, School of Medicine, College of Medicine, Taipei Medical University, Taipei 11031, Taiwan; 4 Graduate Institute of Cancer Biology and Drug Discovery, College of Medical Science and Technology, Taipei Medical University, Taipei 11031, Taiwan; 5 Ph.D. Program for Cancer Molecular Biology and Drug Discovery, College of Medical Science and Technology, Taipei Medical University, Taipei 11031, Taiwan; 6 TMU Research Center of Cancer Translational Medicine, Taipei Medical University, Taipei 11031, Taiwan; 7 Cancer Center, Wan Fang Hospital, Taipei Medical University, Taipei 11696, Taiwan

**Keywords:** bioinformatics, drug repurposing, gene signature, histone deacetylase inhibitor, pancreatic ductal adenocarcinoma

## Abstract

Pancreatic ductal adenocarcinoma (PDAC) is still one of the most aggressive and lethal cancer types due to the late diagnosis, high metastatic potential, and drug resistance. The development of novel therapeutic strategies is urgently needed. *KRAS* (Kirsten rat sarcoma 2 viral oncogene homolog) is the major driver mutation gene for PDAC tumorigenesis. In this study, we mined cancer genomics data and identified a common *KRAS*-driven gene signature in PDAC, which is related to cell–cell and cell–extracellular matrix (ECM) interactions. Higher expression of this gene signature was associated with poorer overall survival of PDAC patients. Connectivity Map (CMap) analysis and drug sensitivity profiling predicted that a clinically approved JAK2 (Janus kinase 2)-selective inhibitor, fedratinib (also known as TG-101348), could reverse the *KRAS*-driven gene signature and exhibit *KRAS*-dependent anticancer activity in PDAC cells. As an approved treatment for myelofibrosis, the pharmacological and toxicological profiles of fedratinib have been well characterized. It may be repurposed for treating *KRAS*-driven PDAC in the future.

## 1. Introduction

The occurrence of pancreatic cancer has significantly ascended throughout the past decade. Among them, pancreatic ductal adenocarcinoma (PDAC) accounts for most cases of pancreatic cancer, with its survival rate being lower than 8% [1,2]. The death rate is highly correlated with high incidence of metastasis, recurrence rate, and chemoresistance. Due to late diagnosis of most clinical cases, the aggressive type was often accompanied by angiogenesis and metastasis, resulting in high unresectable clinical cases [3,4]. Gemcitabine-based chemotherapies, alone or in combination with other drugs such as nab-paclitaxel and FOLFIRINOX (a combination of fluorouracil, leucovorin, irinotecan, and oxaliplatin), are the first-line treatment for locally advanced or metastatic PDAC. However, past clinical results often showed poor prognosis and unsatisfactory drug efficacy [4,5]. Therefore, a more profound knowledge of PDAC biology will help to develop more effective anticancer strategies.

PDAC is usually driven by mutations of the proto-oncogene and tumor suppressor genes, such as *KRAS* (Kirsten rat sarcoma 2 viral oncogene homolog), *TP53* (tumor protein p53), *SMAD4* (SMAD family member 4), *CDKN2A* (cyclin dependent kinase inhibitor 2A), and others [6,7]. Because *KRAS* is the most common mutated driver gene in PDAC, it is considered an ideal therapeutic target. However, *KRAS* remains undruggable for the past three decades due to the failure of the development of effective *KRAS* inhibitors [8]. A breakthrough is the development of *KRAS*^G12C^ (glycine 12 to cysteine)-specific inhibitors, MRTX849 and AMG-510 [9,10]. At the end of 2019, the latter has been granted a fast track designation by the United States Food and Drug Administration (FDA) for treating metastatic non-small-cell lung carcinoma with the *KRAS^G12C^* mutation [11]. Another exciting drug is the first oral pan-*KRAS* inhibitor, BI-1701963, which has been in a phase I clinical trial alone or in combination with the MEK (mitogen-activated protein/extracellular signal-regulated kinase) inhibitor, trametinib, for *KRAS*-mutated solid tumors (NCT04111458; https://clinicaltrials.gov/). The successes of *KRAS* inhibitors make targeting *KRAS*-mutated PDAC possible in the near future.

In this study, we mined bioinformatics resources and identified a common PDAC gene signature that was driven by *KRAS*, but not by *TP53*, mutation. This gene signature was associated with the regulation of cell–cell and cell–extracellular matrix (ECM) interactions. The reversion of this gene signature by a clinically approved JAK2 (Janus kinase 2) inhibitor, fedratinib (also known as TG-101348), may provide therapeutic benefit for *KRAS*-mutated PDAC patients.

## 2. Materials and Methods

### 2.1. Preparation of the Differentially Expressed Genes

The microarray data sets (GSE15471 [12,13], GSE16515 [14,15,16], GSE32676 [17,18], GSE62452 [19], and GSE101448 [20]) containing normal and cancerous pancreatic tissue samples were obtained from the Gene Expression Omnibus (GEO) database at the National Center for Biotechnology Information (NCBI) [21]. The differentially expressed genes (DEGs) were prepared using the R-based web application, GEO2R [21]. The Venn diagram was generated using the InteractiVenn (http://www.interactivenn.net/) [22]. The heat map was generated using the Morpheus (https://software.broadinstitute.org/morpheus).

### 2.2. Pathway Enrichment and Gene Set Enrichment Analysis

Pathway enrichment was performed using the WebGestalt (http://www.webgestalt.org/) [23] and STRING (http://string-db.org/) [24] web-based tools. For WebGestalt analysis, the gene set enrichment analysis (GSEA) method was used to analyze the following functional databases: Gene Ontology (GO) biological processes [25,26], Kyoto Encyclopedia of Genes and Genomes (KEGG) pathways [27], and cancer hallmarks [28]. For STRING analysis, the settings were as follows: active interaction source = experiments and databases; minimum required interaction score = medium confidence (0.400); and max number of interactors to show = none. The enrichment of the PDAC gene signature in these microarray data sets (GSE33323 [29], GSE58055 [30], GSE53659 [31], GSE67358 [32], GSE123646 [33]) was performed using the GSEA v3.0 software (https://www.gsea-msigdb.org/gsea/ [34,35]).

### 2.3. Cancer Genomics Analysis via the cBioPortal Website

The cBioPortal (http://www.cbioportal.org/) is a website to access, analyze, and visualize the large-scale TCGA (The Cancer Genome Atlas) cancer genomics data sets or other studies [36,37]. The “Pancreatic adenocarcinoma (TCGA, PanCancer Atlas)” dataset of 168 PDAC patients containing complete genetic status (mutation, copy number variation, and mRNA expression) was used in this study to compare the association between gene mutations and PDAC gene signature. In addition, a Kaplan–Meier survival plot was generated using the cBioPortal to investigate the impact of PDAC gene signature on patients’ overall survival.

### 2.4. Connectivity Map Analysis

The Connectivity Map (CMap; https://clue.io/) database contains numerous gene signatures from cultured human cancer cell lines treated with drugs [38]. It is believed that a drug has the potential for treating a disease if this drug could reverse the disease-associated gene signature [39,40]. To identify the potential drugs to reverse PDAC gene signature, the commonly upregulated 53 genes were inputted to query the CMap database. The results were visualized as a heat map with a connectivity score between −100 and 100 corresponding to the magnitude of dissimilarity and similarity between queried and existing gene signatures.

### 2.5. Drug Sensitivity Profiling in Pancreatic Ductal Adenocarcinoma Cell Lines

The correlations between *KRAS* gene expression and drug sensitivity in PDAC cancer cell lines were obtained from the CellMinerCDB (https://discover.nci.nih.gov/cellminercdb/ [41]). The Cancer Therapeutics Response Portal (CTRP [42,43,44]) data from the Broad Institute of Massachusetts Institute of Technology (MIT) and Harvard (Cambridge, MA, USA) were used.

## 3. Results

### 3.1. Identification of a Common Gene Signature in Human Pancreatic Ductal Adenocarcinoma

**Figure 1:**
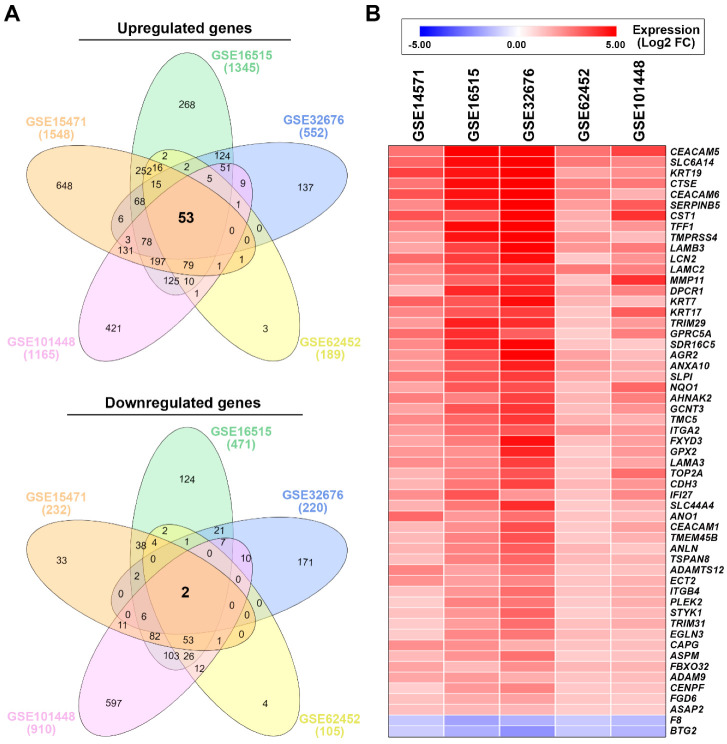
The common gene signature in human pancreatic ductal adenocarcinoma. (**A**) The Venn diagrams show the overlapped gene numbers among five microarray data sets. (**B**) The heat map shows the relative expression for the common gene signature.

**Figure 2:**
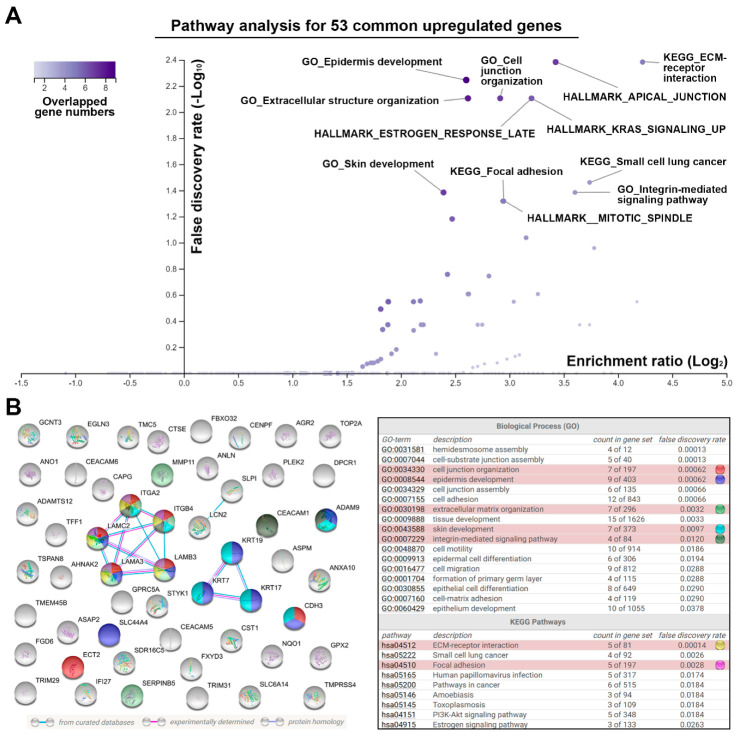
Pathway enrichment for the common upregulated genes in pancreatic ductal adenocarcinoma by the WebGestalt (**A**) and STRING database (**B**) web-based tools. Inset at top left in (**A**): a gradient color key shows the overlapped gene numbers in a pathway. In the volcano plot of (**A**), the purple circles for HALLMARK_ESTROGEN_RESPONSE_LATE/HALLMARK_KRAS_ SIGNALING_UP or KEGG_Focal adhesion/HALLMARK_MITOTIC_SPINDLE were overlapped. In the left part of (**B**), line colors indicate the types of interaction evidence. The cyan and pink lines indicate protein–protein interactions from curated and experimental data, respectively. The purple line indicates that two protein molecules share structural homology. Functional enrichment (gene ontology (GO) biological processes and Kyoto Encyclopedia of Genes and Genomes (KEGG) pathways) in this network are shown in the right part of (**B**). Selected GO biological processes and KEGG pathways are highlighted with different colors. The term “count in gene set” indicates the overlapped genes (the first number) in a pathway (the second number). The term “false discovery rate” indicates the average rate of false coverage for the functional enrichment.

To identify the common gene signature associated with PDAC, five microarray data sets (Table 1) were obtained from the NCBI-GEO database [21]. Then, the DEGs were prepared using the R-based web application, GEO2R [21]. These DEGs (Supplementary Material, File S1) were analyzed using the InteractiVenn web-based tool [22]. As shown in the Venn diagrams (Figure 1A), we identified 53 upregulated and 2 downregulated genes that were common in PDAC tissues when compared with the adjacent normal tissues (Figure 1A). Their expression levels are listed in Table 2 and visualized in a heat map (Figure 1B). To investigate the potential role of this common gene signature, pathway enrichment for the 53 upregulated genes was performed using the WebGestalt web-based tool [23] against GO biological processes [25,26], KEGG pathways [27], and cancer hallmarks [28]. We found that pathways related to cell–cell and cell–ECM interactions were significantly enriched, such as KEGG_ECM-receptor interaction, KEGG_Focal adhesion, HALLMARK_APICAL_JUNCTION, GO_Cell junction organization, GO_Integrin-mediated signaling pathway, and GO_Extracellular structure organization (Figure 2A). The network for the 53 upregulated genes was further constructed and functional enrichment was performed for GO biological processes and KEGG pathways using the STRING database [24]. As shown in Figure 2B, *ITGA2*, *ITGB4*, *LAMA3*, *LAMC2*, *LAMB3*, and *GPRC5A* genes formed a major cluster, which participated in ECM-receptor interaction, focal adhesion, cell junction organization (together with *CDH3* and *ECT2* genes), and extracellular organization (together with *SERPINB5* and *MMP11* genes). Therefore, the alteration of genes related to cell–cell and cell–ECM interactions is a common gene signature in PDAC.

### 3.2. The Pancreatic Ductal Adenocarcinoma Gene Signature Was Associated with KRAS and TP53 Gene Mutations

**Figure 3:**
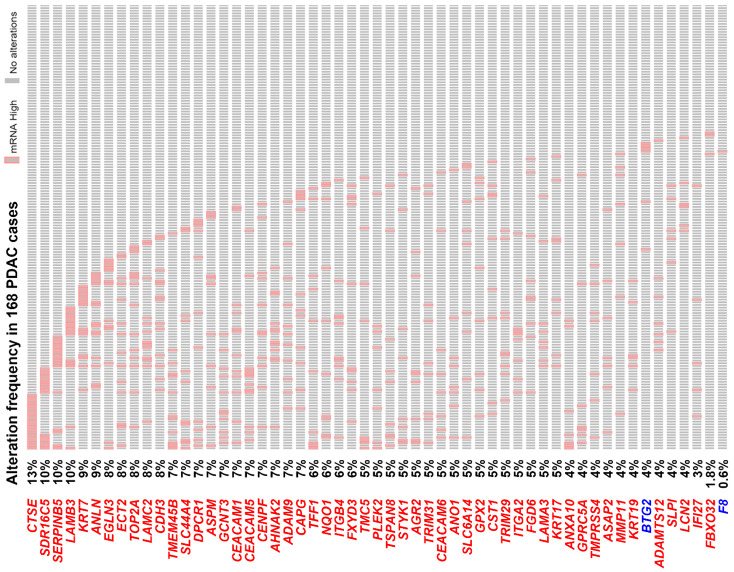
A waterfall plot for the common gene signature expression in “The Cancer Genome Atlas—Pancreatic adenocarcinoma” data set. Genes highlighted in red or blue color indicate those commonly upregulated or downregulated in pancreatic ductal adenocarcinoma (PDAC) patients, respectively. The cases highlighted in red grids (labeled as “mRNA High”) indicate those with mRNA expression higher than that of the average patient (z-score >2).

**Figure 4:**
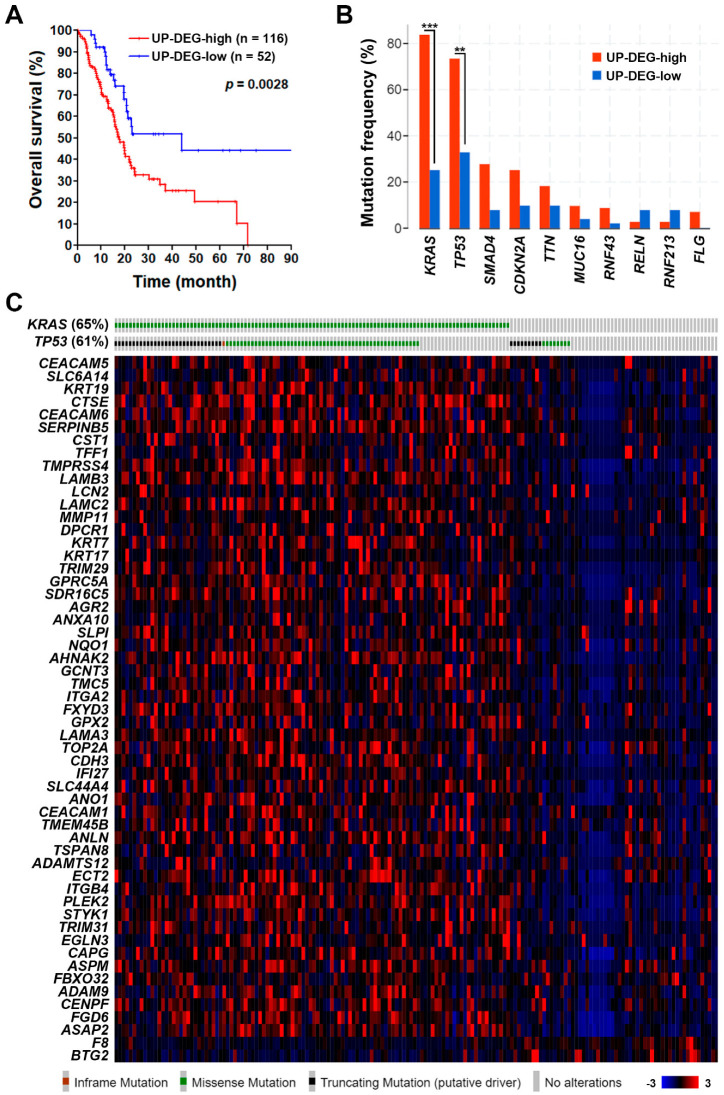
The association of pancreatic ductal adenocarcinoma gene signature with patients’ overall survival and gene mutations. (**A**) A Kaplan–Meier plot shows the association of 53 common upregulated genes (UP-DEG) with PDAC patients’ overall survival. The term “UP-DEG-high” indicates patients with higher mRNA expression (z-score >2) of any one of the 53 common upregulated genes. The remaining patients are classified as “UP-DEG-low” cases. (**B**) The association of 53 common upregulated genes (UP-DEG) with PDAC patients’ gene mutation status. (**C**) A heat map shows the association of PDAC gene signature with *KRAS* and *TP53* mutations. Inset at bottom right: a gradient color key shows the related gene z-scores.

To further confirm the role of PDAC gene signature, the TCGA-PAAD (pancreatic adenocarcinoma) data set with 168 PDAC cases was used to compare their mRNA levels. As shown in Figure 3, most of them have higher mRNA expressions, especially for the 10 genes related to cell–cell and cell–ECM interactions: *LAMB3* (10%), *SERPINB5* (10%), *CDH3* (8%), *ECT2* (8%), *LAMC2* (8%), *ITGB4* (6%), *ITGA2* (5%), *LAMA3* (5%), *GPRC5A* (4%), and *MMP11* (4%). PDAC patients with the higher gene signature (53 upregulated genes only) expression have poorer overall survival (Figure 4A). In addition, we found that the upregulation of PDAC gene signature was significantly associated with *KRAS* and *TP53* gene mutations (Figure 4B,C). According to the TCGA-PAAD data set, 110 (65%) and 102 (61%) of the 168 PDAC cases harbored *KRAS* and *TP53* gene mutations, respectively (Figure 4B). Given the fact that *KRAS* and *TP53* were the most two common mutated genes in PDAC [6,7], it is reasonable that the PDAC gene signature may be driven by *KRAS* and *TP53* gene mutations during tumorigenesis.

### 3.3. Gene Set Enrichment Analysis Revealed That the Pancreatic Ductal Adenocarcinoma Gene Signature Was Driven by KRAS Gene Mutation

**Figure 5:**
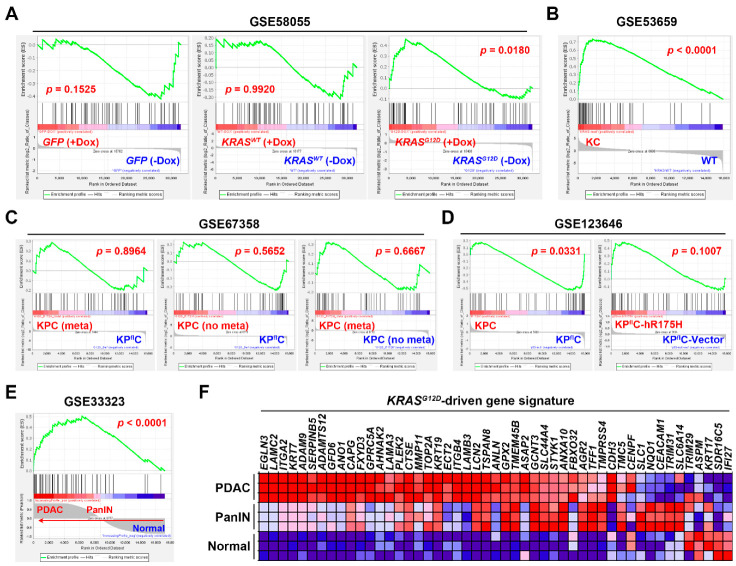
The association between *KRAS*/*TP53* gene mutations and pancreatic ductal adenocarcinoma gene signature. Gene set enrichment analysis (GSEA) was performed to enrich the PDAC gene signature in the following data sets. (**A**) GSE58055: HPDE-E6/E7 cells stably transfected with doxycycline (Dox)-inducible *GFP* (left part), *KRAS^WT^* (middle part), and *KRAS^G12D^* (right part). (**B**) GSE53659: *KRAS^G12D^*-driven PDAC cells from KC (*Pdx1-Cre*/*Kras^G12D/+^*) mice compared with normal pancreatic cells from WT mice. (**C**) GSE67358: The metastatic (meta) and non-metastatic (no meta) PDAC cells from KPC (*Pdx1-Cre*/*Kras^G12D/+^*/*Trp53^R172H/+^*) mice were compared with the PDAC cells from KP^fl^C (*Pdx1-Cre*/*Kras^G12D/+^*/*Trp53^-/+^*) mice (left and middle parts) and with each other (right part). (**D**) GSE123646: In the left part, PDAC cells from KPC mice irrespective of their metastatic status were compared with those from KP^fl^C mice. In the right part: KP^fl^C PDAC cells transfected with a plasmid encoding human *TP53^R175H^* were compared with those transfected with a control vector. (**E**) GSE33323: Normal, pancreatic intraepithelial neoplasia (PanIN), and PDAC tissues from KC mice were compared. Notes for (**A**–**E**): The top portion of an enrichment plot shows the running enrichment score (ES) for the gene set (53 PDAC signature genes) as the analysis walks down the ranked list (as indicated by a green line). The ES is the maximum deviation from zero encountered in walking down the list. A positive or negative ES indicates gene set enrichment at the top or bottom of the ranked list, respectively. The bottom portion shows the ranking metric scores (as indicated by the grey graph) that represent a gene’s correlation with a phenotype (such as a treatment). For categorical phenotypes in (**A**–**D**), the metric “Log2_Ratio_of_ Classes” was used to calculate fold changes (Log2 ratio) for gene expression differences between two phenotypes. A positive or negative value indicates the correlation of the gene set with the first or second phenotype, respectively. For continuous phenotypes (Normal → PanIN → PDAC) in (**E**), the Pearson’s correlation metric was used. A positive value indicates the correlation of the gene set with the phenotype profile and a negative value indicates no correlation or inverse correlation of the gene set with the phenotype profile. The middle portion is a barcode plot showing the position of 53 PDAC signature genes (denoted as “Hits”) in the ranked list. The “zero cross” (a dash line) indicates the point at which the calculated difference between expression in two or continuous phenotypes is 0. Red or blue gradient colors around the “zero cross” correspond to the expression levels of the ranked list. Genes with the darker red or blue are expressed higher in the first or second phenotype, respectively. (**F**) The heat map for the relative expression of PDAC gene signature in GSE33323 microarray data set.

To further investigate whether the PDAC gene signature was driven by *KRAS* and *TP53* gene mutations, the effect of *KRAS^G12D^* (glycine 12 to aspartate) or *TP53^R175H^* (arginine 175 to histidine; the human equivalent of mouse *Trp53^R172H^*) mutations on PDAC gene signature was analyzed by GSEA using the relevant microarray data sets (Table 3). In GSE58055 [30], a doxycycline (Dox)-inducible *KRAS^G12D^* mutation was introduced into the E6/E7-transformed human pancreatic ductal epithelial (HPDE) cells, in which the E6 and E7 proteins of the HPV16 virus inactivate p53 and RB, respectively [45,46]. We found that the PDAC gene signature was only enriched in HPDE cells with *KRAS^G12D^* induction by Dox, but not in cells with the induction of wild-type (WT) *KRAS* or green fluorescent protein (*GFP*) control vector (Figure 5A), suggesting that the PDAC gene signature can be driven by *KRAS^G12D^* mutation. To confirm the above observation, another microarray data set GSE53659 [31] with the *Kras^G12D^*-driven PDAC in mice (*Pdx1-Cre*/*Kras^G12D/+^*; also known as KC mice) was used. As shown in Figure 5B, the PDAC gene signature was enriched in *KRAS^G12D^*-driven PDAC cells compared with that in the WT cells. Therefore, the PDAC gene signature is indeed driven by *KRAS^G12D^* mutation.

To investigate the role of *TP53* (*Trp53* in mice) gene mutation, two microarray data sets, GSE67358 [32] and GSE123646 [33], were employed. It has been shown that one-third of KC mice develop PDAC by 500 days [47], and the additional *Trp53* mutation in KPC (*Pdx1-Cre*/*Kras^G12D/+^*/*Trp53^R172H/+^*) mice or *Trp53* deletion in KP^fl^C (*Pdx1-Cre*/*Kras^G12D/+^*/*Trp53^-/+^*) mice accelerates the tumor development by 120–180 days [48]. However, only Trp53 mutation, but not deletion, can drive tumor metastasis in this model [49], suggesting a synergy between *KRAS* and *TP53* mutations to promote PDAC progression. Because the incidence of tumor metastasis is about 65% in KPC mice [49], the gene expression profiles of both metastatic (meta) and non-metastatic (no meta) PDAC cells from KPC mice were compared with that from KP^fl^C mice (Figure 5C, the left and middle parts). In addition, the gene expression profiles of metastatic and non-metastatic PDAC cells were also compared to each other (Figure 5C, the right part). We found that the PDAC gene signature was not enriched in any group, suggesting that the PDAC gene signature was not associated with *TP53* (*Trp53*) mutation and its metastasis-promoting effect. It was puzzling that inconsistent observation was found in the GSE123646 data set (Figure 5D). The PDAC gene signature was enriched in KPC mice-derived PDAC cells (irrespective of their metastatic status) compared with that in KP^fl^C mice-derived PDAC cells. However, the PDAC gene signature was not enriched in KP^fl^C mice-derived PDAC cells transfected with the human equivalent of murine *Trp53^R172H^* (*TP53^R175H^*). Such discrepancy may imply the minimal effect of *TP53* gene mutation on PDAC gene signature expression, which warrants further investigation.

The above results argue for the essential role of *KRAS* gene mutation in PDAC gene signature expression. To further investigate the expression of PDAC gene signature during *KRAS^G12D^*-driven pancreatic tumorigenesis, the gene expression profiles of normal pancreas, pancreatic intraepithelial neoplasia (PanIN) and PDAC in KC mice were obtained from the microarray data set GSE33323 [29]. GSEA showed that the PDAC gene signature is significantly correlated with PanIN and PDAC (Figure 5E). The related expression of PDAC gene signature was visualized in a heat map showing that the PDAC gene signature was induced during *KRAS^G12D^*-driven PDAC development in KC mice (Figure 5F). Taken together, we conclude that the PDAC gene signature is driven by *KRAS*, but not *TP53*, gene mutation.

### 3.4. Connectivity Map Analysis and Drug Sensitivity Profiling Identify TG-101348 (Fedratinib) as a Potential Drug Reversing KRAS-Driven Pancreatic Ductal Adenocarcinoma Gene Signature

**Figure 6:**
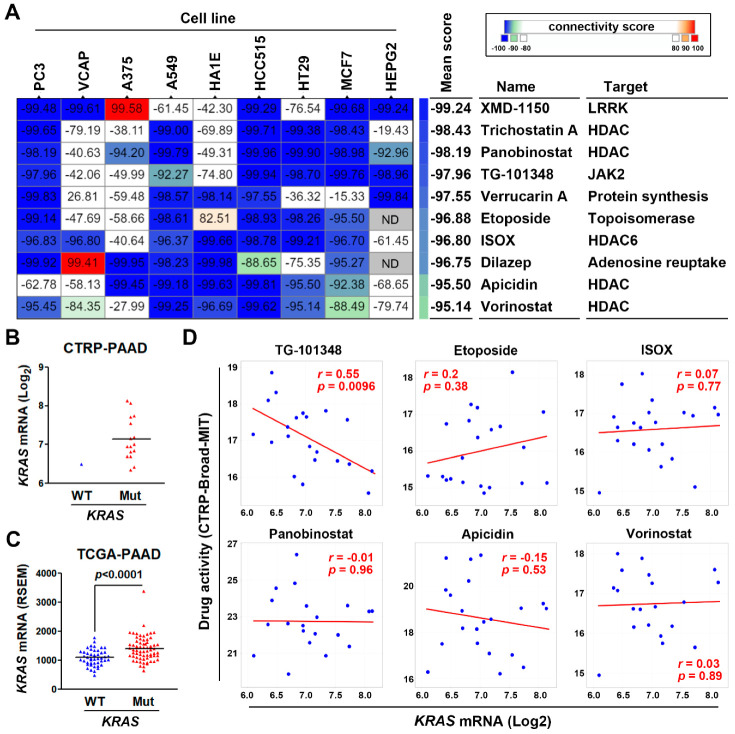
Connectivity Map analysis and drug sensitivity profiling in pancreatic ductal adenocarcinoma cells. (**A**) The PDAC gene signature (53 upregulated genes) was queried using the CMap database to predict potential drugs to reverse this signature. The connectivity scores ranging from −100 to 100 correspond to the dissimilarity and similarity between the queried and existing gene signatures in each drug-treated cancer cell line. Inset at top right: a color key shows the connectivity scores. The blue and red colors indicate the scores of −100 and 100, respectively. (**B**) The correlation between *KRAS* gene mutation and mRNA levels in PDAC cell lines (The Cancer Therapeutics Response Portal CTRP-PAAD (pancreatic adenocarcinoma) data from the CTRP database). (**C**) The correlation between *KRAS* gene mutation and mRNA levels in PDAC cancer tissues (The Cancer Genome Atlas (TCGA)-PAAD data from the cBioPortal database). (**D**) The correlation between drug activity and *KRAS* mRNA levels in PDAC cell lines (CTRP-PAAD data from the CTRP database).

To identify potential drugs that could reverse the *KRAS*-driven PDAC gene signature, we employed the CMap database that contains numerous gene signatures from cultured human cancer cell lines treated with drugs [38]. If a drug could reverse a disease-associated gene signature, this drug is believed to have the potential to cure the disease [39,40]. We queried the CMap database with the PDAC gene signature (53 upregulated genes) to connect the PDAC gene signature to drug-derived gene signatures. The CMap connectivity score ranging from −100 to 100 corresponds to the magnitude of dissimilarity and similarity between queried and existing gene signatures. Figure 6A showed the most dissimilar drugs (connectivity score < −95) representing the potential drugs that could reverse the queried PDAC gene signature. Interestingly, most of them belong to histone deacetylase (HDAC) inhibitors including trichostatin A (pan-HDAC), panobinostat (pan-HDAC), ISOX (HDAC6-specific), apicidin (pan-HDAC), and vorinostat (pan-HDAC). Therefore, inhibition of HDAC might have the potential to treat PDAC by reversing its *KRAS*-driven gene signature.

We hypothesized that a drug may exhibit *KRAS*-dependent cytotoxicity in cancer cells if this drug could reverse the *KRAS*-driven gene signature. To examine whether the predicted CMap drugs could exhibit *KRAS*-dependent cytotoxicity in PDAC cells, we employed the CellMinerCDB database that is a web-based tool enabling to explore and analyze pharmacological and genomic data of human cancer cell lines [41]. Due to the frequent *KRAS* mutation in PDAC, there was only one PDAC cell line (BxPC-3) harboring the wild-type *KRAS* gene (Figure 6B). Thus, it is impossible to correlate the drug activity to *KRAS* gene mutation. According to the gene expression profiles from PDAC cell lines (CTRP-PAAD) and patients’ tissues (TCGA-PAAD; Figure 6C), the mutant *KRAS* gene tended to be expressed higher compared to the wild-type *KRAS* gene. Thus, as an alternative, we correlated the drug activity with *KRAS* mRNA expression. The CTRP database only contained the drug sensitivity profiles for TG-101348, etoposide, ISOX, panobinostat, apicidin, and vorinostat. Surprisingly, pan-HDAC (panobinostat, apicidin, and vorinostat) and HDAC6 (ISOX) inhibitors, as well as etoposide, did not exhibit *KRAS*-dependent cytotoxicity (Figure 6D). Only TG-101348 displayed significant association with *KRAS* expression (Figure 6D). Therefore, TG-101348 may exhibit *KRAS*-dependent anticancer activity in PDAC cells via the reversion of *KRAS*-driven gene signature.

## 4. Discussion

Dynamic cell–cell and cell–ECM interactions maintain a tumor microenvironment that consists of acellular fibrous stroma and diverse populations of the non-neoplastic cancer-associated cells. Previous studies suggested that the tumor progression of PDAC as well as its deadly malignancy are highly associated with the tumor microenvironment. Thus, targeting the stromal compartment in PDAC may have anticancer effects and enhance chemo-/radio-sensitivity [50,51,52]. Our results imply that inhibition of PDAC gene signature (genes related to cell–cell and cell–ECM interactions) may be beneficial for treating PDAC via a remodeling of the tumor microenvironment.

According to TCGA-PAAD data, there were several *KRAS* mutation types in PDAC patients (Supplementary Material, Figure S1A), including *KRAS^G12C^* (glycine 12 to cysteine; n = 1), *KRAS^G12D^* (n = 45), *KRAS^G12V^* (glycine 12 to valine; n = 31), *KRAS^G12S^* (glycine 12 to serine; n = 1), *KRAS^G12A^* (glycine 12 to alanine; n = 1), *KRAS^G12R^* (glycine 12 to arginine; n = 24), *KRAS^G13C^* (n = 1), *KRAS^Q61R^* (glutamine 61 to arginine; n = 2), and *KRAS^Q61H^* (glutamine 61 to histidine; n = 6). The most frequent mutation types were *KRAS^G12D^*, *KRAS^G12V^*, and *KRAS^G12R^*. In this study, only the impact of *KRAS^G12D^* on PDAC gene signature was analysed by GSEA. The roles of other *KRAS* gene mutations were still unclear. However, we found that the expression levels of PDAC gene signature were similar in patients with different *KRAS* mutation types (Supplementary Material, Figure S1B and Table S1). Furthermore, GSEA indicated that the PDAC gene signature was significantly enriched in patients with *KRAS^G12D^*, *KRAS^G12V^*, and *KRAS^G12R^* (Supplementary Material, Figure S1C). Therefore, the PDAC gene signature could be driven by different *KRAS* mutation types.

TG-101348, also known as fedratinib, is a JAK2-selective inhibitor that has been approved for treating patients with myelofibrosis [53]. Myelofibrosis is a rare type of bone marrow cancer, which disrupts the normal production of blood cells. The discovery of *JAK2^V617F^* (valine 617 to phenylalanine) mutation in myelofibrosis uncovers the activated JAK–STAT (signal transducer and activator of transcription) signaling as a primary driver for myelofibrosis, and supports the rationale for treating myelofibrosis by JAK2 inhibition [54]. Interestingly, a previous study has shown that STAT3 plays a critical role in *KRAS*-induced PDAC tumorigenesis. A large-scale cancer cell line screening identified a JAK2-selective inhibitor, AZ960, that blocks STAT3 activation and exhibits higher sensitivity against PDAC cell lines [55], which supports the utility of therapeutic targeting of JAK2–STAT3 signaling in PDAC.

HDAC inhibitors have been viewed as a prominent class of therapeutic agents for treating PDAC [56,57]. However, the impact of *KRAS* mutation on their anticancer activity was largely unclear. Our results suggest that the anticancer activity of HDAC inhibitors is unrelated to *KRAS* mutation status. Consistently, previous studies found that pan-HDAC inhibitors (vorinostat and AR-42) exhibit similar cytotoxicity in both *KRAS* WT and mutant PDAC cells [58,59]. However, it was also reported that a HDAC inhibitor, romidepsin, preferentially induces apoptosis in cancer cells harboring mutant *KRAS* [60]. More investigations are needed to clarify the exact role of *KRAS* mutation in the anticancer activity of HDAC inhibitors.

## 5. Conclusions

This study integrates bioinformatics resources to investigate the key driver mutation gene, *KRAS*, and the associated gene signature in PDAC. Our results demonstrate that the progression and prognosis of PDAC is highly associated with a *KRAS*-driven gene signature related to cell–cell and cell–ECM interactions. A FDA-approved JAK2-selective inhibitor, fedratinib (TG-101348), is predicted to reverse the *KRAS*-driven gene signature, thereby providing therapeutic benefit for *KRAS*-mutated PDAC patients.

## Figures and Tables

**Table 1:** Microarray data sets from human pancreatic cancer patients.

Access Number	Platform	# of Cases		# of DEGs ^1^		References
		Normal	Tumor	Up	Down	
GSE15471	HG-U133_Plus_2 ^2^	39	39	1548	232	[12,13]
GSE16515	HG-U133_Plus_2	16	36	1345	471	[14,15,16]
GSE32676	HG-U133_Plus_2	7	25	552	220	[17,18]
GSE62452	HG-U133_Plus_2	61	69	189	105	[19]
GSE101448	Illumina_HT-12_V4 ^3^	19	24	1165	910	[20]

^1^ Differentially expressed genes (DEGs): adjusted *p* value <0.05 and fold change (FC) >1. ^2^ Affymetrix Human Genome U133 Plus 2.0 Array. ^3^ Illumina HumanHT-12 V4.0 expression BeadChip.

**Table 2:** The common gene signature in pancreatic ductal adenocarcinoma and the gene FC values.

	GSE15471	GSE16515	GSE32676	GSE62452	GSE101448	Average FC
*CEACAM5*	2.77	6.25	6.81	2.79	3.68	4.46
*SLC6A14*	3.01	4.63	5.88	2.66	2.39	3.71
*KRT19*	3.71	4.47	6.22	1.83	2.22	3.69
*CTSE*	2.73	4.62	5.12	2.55	2.69	3.54
*CEACAM6*	3.35	4.53	5.79	2.42	1.56	3.53
*SERPINB5*	2.32	4.39	5.62	1.97	3.22	3.50
*CST1*	3.35	3.04	4.90	1.65	3.96	3.38
*TFF1*	2.40	4.68	5.06	1.51	2.12	3.15
*TMPRSS4*	1.96	4.50	5.76	2.06	1.36	3.13
*LAMB3*	1.79	3.67	4.87	2.08	2.64	3.01
*LCN2*	2.89	3.79	4.59	1.12	2.15	2.91
*LAMC2*	2.18	3.55	3.59	2.65	2.51	2.90
*MMP11*	2.05	3.05	4.16	1.23	3.99	2.90
*DPCR1*	1.35	4.15	4.23	1.78	2.47	2.80
*KRT7*	3.11	3.29	4.65	1.49	1.34	2.77
*KRT17*	2.38	3.27	3.77	1.18	3.20	2.76
*TRIM29*	2.00	4.30	4.06	1.28	2.01	2.73
*GPRC5A*	2.85	4.05	3.14	1.01	2.53	2.72
*SDR16C5*	2.32	4.18	4.78	1.20	1.07	2.71
*AGR2*	2.05	3.37	4.83	1.86	1.36	2.69
*ANXA10*	2.01	3.25	4.32	1.94	1.70	2.64
*SLPI*	2.67	3.31	3.79	1.73	1.61	2.62
*NQO1*	1.80	3.28	3.45	1.31	2.98	2.56
*AHNAK2*	2.54	2.48	3.71	1.51	2.54	2.56
*GCNT3*	1.85	3.35	3.93	1.34	2.15	2.52
*TMC5*	2.36	3.00	3.86	1.55	1.55	2.46
*ITGA2*	2.00	2.83	3.29	2.14	2.02	2.46
*FXYD3*	1.80	2.59	4.57	1.32	1.91	2.44
*GPX2*	2.07	2.18	4.20	1.07	2.01	2.31
*LAMA3*	2.26	2.33	3.75	1.24	1.81	2.28
*TOP2A*	1.51	2.46	3.36	1.16	2.86	2.27
*CDH3*	1.50	2.68	3.65	1.43	2.07	2.27
*IFI27*	2.24	3.33	2.10	1.23	2.36	2.25
*SLC44A4*	1.56	2.68	4.06	1.08	1.55	2.18
*ANO1*	2.97	2.03	2.83	1.20	1.36	2.08
*CEACAM1*	1.42	2.24	3.47	1.10	1.84	2.01
*TMEM45B*	1.41	2.49	3.39	1.12	1.54	1.99
*ANLN*	1.52	2.44	3.18	1.47	1.15	1.95
*TSPAN8*	1.30	2.48	3.02	1.39	1.49	1.94
*ADAMTS12*	2.42	1.87	2.69	1.14	1.25	1.87
*ECT2*	2.18	1.93	2.40	1.19	1.55	1.85
*ITGB4*	1.23	2.09	2.87	1.23	1.63	1.81
*PLEK2*	1.01	2.47	2.71	1.09	1.64	1.78
*STYK1*	1.25	2.08	3.05	1.03	1.42	1.77
*TRIM31*	1.06	1.97	2.84	1.27	1.68	1.76
*EGLN3*	1.06	2.38	2.70	1.39	1.25	1.76
*CAPG*	2.23	2.23	1.62	1.22	1.30	1.72
*ASPM*	1.38	2.17	2.80	1.03	1.21	1.72
*FBXO32*	1.82	1.39	2.21	1.45	1.51	1.68
*ADAM9*	1.76	2.00	1.65	1.20	1.33	1.59
*CENPF*	1.00	2.03	2.44	1.12	1.24	1.57
*FGD6*	1.26	1.68	1.87	1.18	1.07	1.41
*ASAP2*	1.27	1.44	1.47	1.03	1.04	1.25
*F8*	−1.11	−1.83	−1.56	−1.07	−1.37	−1.39
*BTG2*	−1.02	−1.61	−2.17	−1.12	−1.51	−1.49

**Table 3:** Microarray data sets from *KRAS*-mutant and *TP53* (*Trp53*)-mutant cells.

Access Number	Platform	Samples	Reference
GSE58055	Agilent SurePrint G3 Human Gene Expression 8x60K v2 Microarray	Immortalized HPDE-E6/E7 cells stably transfected with a doxycycline (Dox)-inducible *KRAS^WT^* (n = 4) or *KRAS^G12D^* (n = 6) plasmid, or a control *GFP* vector (n = 6).	[30]
GSE53659	Affymetrix Mouse Genome 430 2.0 Array	Normal pancreas from WT mice (n = 5); PDAC cells from KC (*Pdx1-Cre*/*Kras^G12D/+^*) mice (n = 6)	[31]
GSE67358	Affymetrix Mouse Genome 430 2.0 Array	Metastatic (n = 7) and non-metastatic (n = 7) PDAC cells from KPC (*Pdx1-Cre*/*Kras^G12D/+^*/*Trp53^R172H/+^*) mice; PDAC cells (n = 5) from KP^fl^C (*Pdx1-Cre*/*Kras^G12D/+^*/*Trp53^-/+^*) mice.	[32]
GSE123646	Affymetrix Mouse Genome 430 2.0 Array	KPC (n = 3) and KP^fl^C (n = 3) PDAC cells; KP^fl^C PDAC cells transfected with either a human *TP53^R175H^* plasmid (n = 3) or a control vector (n = 3).	[33]
GSE33323	Affymetrix Mouse Gene 1.0 ST Array	Normal pancreas (n = 3), pancreatic intraepithelial neoplasia (PanIN; n = 3) and PDAC (n = 3) from KC mice	[29]

## Supplementary Materials

The following are available online at https://www.mdpi.com/2075-4426/10/3/130/s1, Table S1: The correlation between *KRAS* mutation types and PDAC gene signature, Figure S1: Role of *KRAS* mutation types in PDAC gene signature expression, Figure S1A: Visualization of the *KRAS* mutation burden and hotspots in 168 PDAC patients, Figure S1B: A heat map shows the correlation between *KRAS* mutation types and PDAC gene signature expression, Figure S1C: GSEA results for the role of *KRAS* mutation types in regulating PDAC gene signature, File S1: The differentially expressed genes (DEGs) prepared from microarray data sets.
