# Supplementary material for: Bioinformatics Data Mining Repurposes the JAK2 (Janus Kinase 2) Inhibitor Fedratinib for Treating Pancreatic Ductal Adenocarcinoma by Reversing the KRAS (Kirsten Rat Sarcoma 2 Viral Oncogene Homolog)-Driven Gene Signature
Source: J Pers Med. 2020 Sep 16;10(3):130. doi: 10.3390/jpm10030130 (PMC7563462; doi:10.3390/jpm10030130)
Supplement: Supplementary file 1 [file jpm-10-00130-s001.zip › Figure S1.pdf]

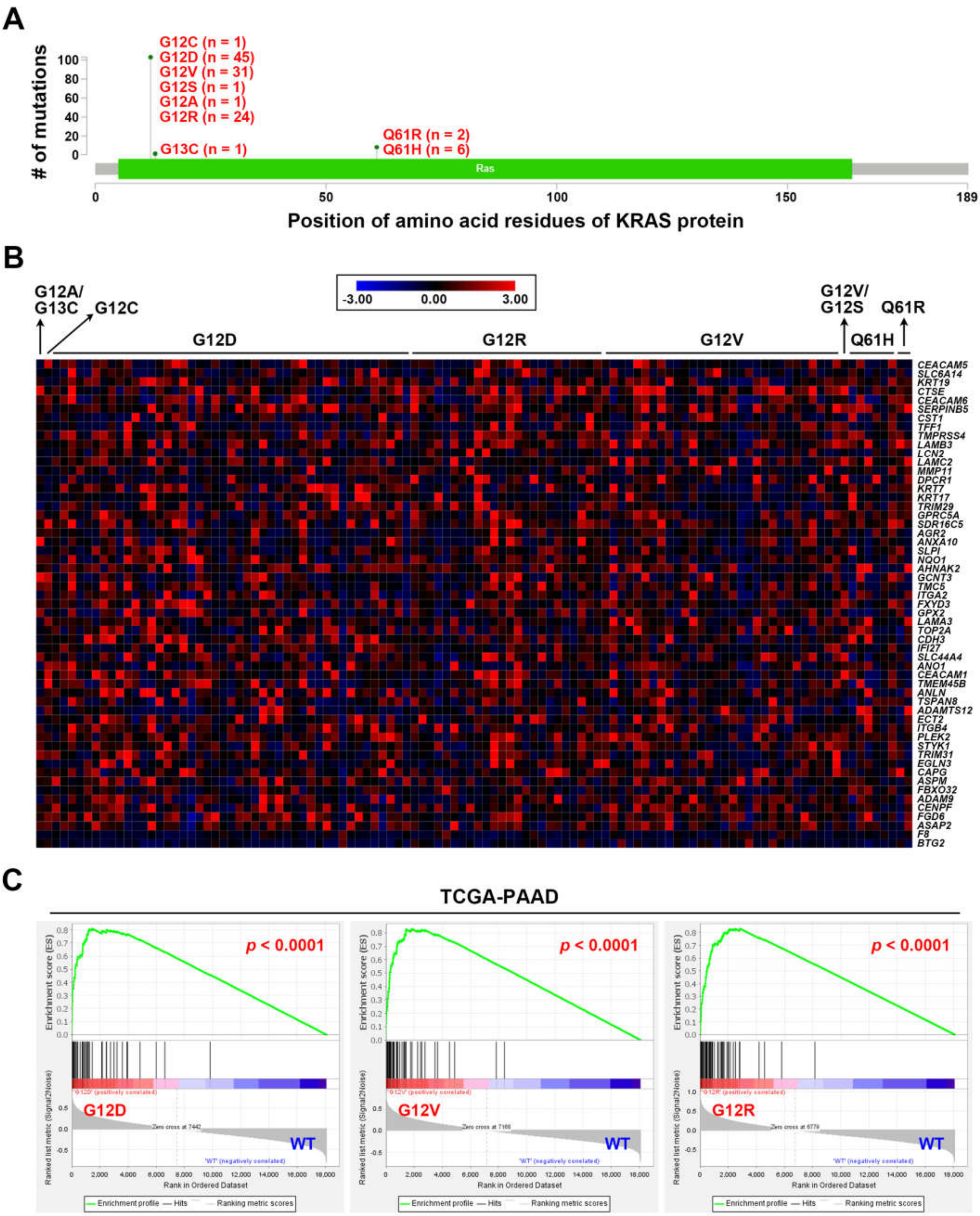

Figure S1. Role of *KRAS* mutation types in PDAC gene signature expression. **(A)** Visualization of the *KRAS* mutation burden and hotspots in 168 PDAC patients. The data were obtained from the "Pancreatic adenocarcinoma (TCGA, PanCancer Atlas)" dataset. **(B)** A heat map shows the correlation between *KRAS* mutation types and PDAC gene signature expression. Inset at top: a gradient color key shows the related gene z-scores. **(C)** GSEA results for the role of *KRAS* mutation types in regulating PDAC gene signature. GSEA was performed to enrich the PDAC gene signature in PDAC patients with $KRAS^{G12D/V/R}$ mutations compared with those with $KRAS^{WT}$.
